# Effect of Alkyl- vs Alkoxy-Arene Substituents on the Deactivation Processes and Fluorescence Quantum Yields of Exciplexes

**DOI:** 10.1021/acs.jpca.4c06279

**Published:** 2024-12-13

**Authors:** Joseph P. Dinnocenzo, Olesya Haze, Cavan Fleming, Samir Farid

**Affiliations:** Department of Chemistry, University of Rochester, Rochester, New York 14627-0216, United States

## Abstract

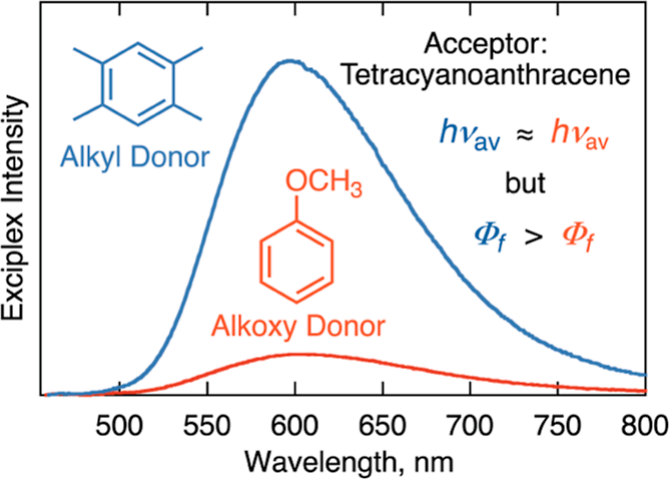

Decay processes of exciplexes of cyanoanthracenes with alkylbenzene donors were compared to those with alkoxybenzenes. For the three decay processes of exciplexes, the radiative rate constant (*k*_f_) of alkoxy derivatives is slightly lower than those of alkylbenzenes at the same average exciplex energy. However, the corresponding deactivation rate constants, intersystem crossing (*k*_isc_) and nonradiative decay (*k*_nr_), are considerably higher. Consequently, the fluorescence quantum yields of the alkoxybenzene exciplexes are one-half to one-fifth of the corresponding alkylbenzenes at comparable emission energies. This trend is solvent-independent. The results can be rationalized in terms of the differing electronic character of the donor radical cation moieties in the alkoxy- vs alkylbenzene exciplexes and differences in their reorganization energies. The impact of these results for the design of exciplexes that emit more efficiently is discussed.

## 1 Introduction

Exciplexes, composed of mixed locally excited and ion pair configurations (e.g., A*D ↔ A^•–^D^•+^), play an important role in a wide variety of fundamental electron transfer processes and applied materials. For example, exciplexes are key intermediates in photoinduced charge transfer processes, including intra- and intermolecular electron transfers in solution^1−5^ and in biological systems (e.g., DNA^6−9^ and photosynthetic reaction centers^10^). Since their initial discovery,^11^ a number of properties of exciplex emission have been probed, including their temperature dependence,^12−15^ effects of medium polarity,^16−18^ their formation in polymer matrices^19−24^ and confined environments,^25,26^ their electronic coupling and reorganization energies,^27−31^ and their role in electron transfer reactions.^32,33^ The photophysical properties of emissive exciplexes have led to their uses in a number of different applications, e.g., as sensors^34−38^ and as probes of biological systems.^8,39−41^ Recently, there has been intense interest in exciplexes in organic light-emitting diodes (OLEDs)^42−46^ and photovoltaic^47−51^ materials. For example, high-efficiency OLED materials based on thermally activated delayed fluorescence (TADF) that utilize exciplexes as key intermediates are under active investigation.^52−55^ The facile tunability of exciplex emission through systematic changes in the donor and acceptor moieties also makes them promising candidates for inclusion in materials for the construction of white organic light-emitting diodes (WOLEDs).^43,56^ In materials for nonlinear photonics, the absorptive properties of the charge transfer species in exciplexes have been found to significantly increase their nonlinear absorption.^57^ Advances in materials and devices for optoelectronics based on exciplex emission have been reviewed.^58^

**Scheme 1:**
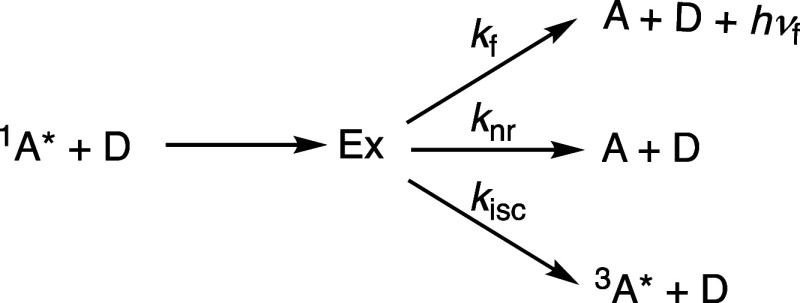
Decay Processes of Exciplexes

To fully take advantage of exciplex emission, it is important to understand factors that affect emission and competing processes. In addition to fluorescence—a radiative return electron transfer process (*k*_f_, Scheme 1)—exciplexes also decay by nonradiative electron transfer to the ground state (*k*_nr_) and via intersystem crossing to the triplet state of one of the reactants (*k*_isc_).

The exciplex fluorescence quantum yield (Φ_f_), eq 1, depends on the ratio of *k*_f_ to *k*_nr_ and *k*_isc_, as shown in eq 1.
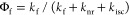
1

Exciplexes with many types of donors and acceptors have been described. With aromatic hydrocarbons as electron acceptors, the donors are usually alkoxy- or amino-substituted aromatic compounds. With cyanoaromatic acceptors, the donors are typically alkyl-substituted aromatics, e.g., alkyl-substituted benzenes.

## 2 Methods

### 2.1 Materials

9,10-Dicyanoanthracene (DCA), 2,9,10-tricyanoanthracene (TriCA), and 2,6,9,10-tetracyanoanthracene (TCA) were available from previous studies.^59^ All electron donors were purified before use by either fractional distillation or recrystallization. All solvents were fractionally distilled before use.

### 2.2 Fluorescence Measurements

Fluorescence spectra were measured using a Fluorolog-3 spectrofluorometer (Jobin Yvon, Horiba) at 20 °C and were corrected for the efficiency of the monochromator and the spectral response of the photomultiplier tube using the Calibration Kit Spectral Fluorescence Standards BAM-F001 to BAM-F005 (Sigma-Aldrich).

Measurements were performed in argon-saturated solutions. To decrease the noise, 12–15 runs were typically averaged to generate the final spectra. There was no discernible difference between the spectra of the individual runs, indicating no degradation or product formation. The optical densities of solutions varied between ∼0.05 and 0.25 depending on the excitation wavelength.

To determine the exciplex fluorescence quantum yields, emission spectra were measured at different excitation wavelengths and corrected for different fractions of absorbed light by dividing the fluorescence intensity by (1–10^–OD^). DCA in air-saturated acetonitrile (ϕ_f_ = 0.80)^60^ was used as an actinometer.

### 2.3 Fluorescence Lifetimes

Fluorescence lifetime measurements were made at 20 °C using the time-correlated single photon counting (TCSPC) method. The output of a tunable (680–1080 nm), 80 MHz femtosecond titanium-sapphire (Ti/S) laser was serially passed through a pulse selector and a harmonic generator. The repetition rate was varied to ensure sufficient decay of excited states between laser pulses. The excitation beam from the second harmonic of the Ti/S wavelength was passed through a Glan–Taylor polarizer to ensure clean vertical polarization and could be attenuated with a rotating neutral filter of variable optical density. The excitation beam entered a FluoTime200 fluorescence lifetime spectrometer equipped with a PicoHarp300 TCSPC module (PicoQuant) and a Hamamatsu R3809U-50 MCP-PMT. The emission beam was passed through a polarizer set at the magic angle. Dilute Ludox solutions were used to collect the instrument response function (IRF) at the excitation wavelength, which had a full width at half-maximum (fwhm) of ∼50 ps. Emission decays were monitored at multiple wavelengths. The fluorescence decays were analyzed using the FluoFit software package (PicoQuant, ver. 4.6.0.0).

### 2.4 Computations

All calculations were carried out with Spartan’20^61^ using the M06 density functional.^62^ Open-shell calculations were performed using the unrestricted UM06 method. Geometry optimizations were performed with the 6-311 + G(2df,2p) basis set.

## 3 Results and Discussion

**Figure 1:**
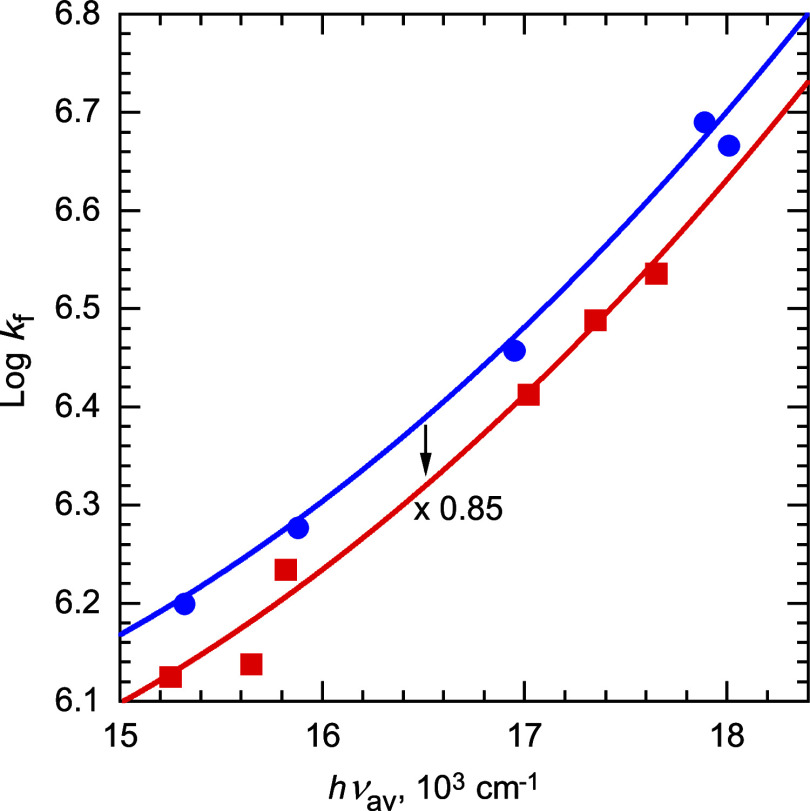
Plot of the logarithm of radiative rate constants, *k*_f_, for cyanoanthracene exciplexes in benzene vs average emission energy, *h*ν_av_. Donors: alkylbenzenes (blue) and alkoxybenzenes (red); data from Table 1.

**Figure 2:**
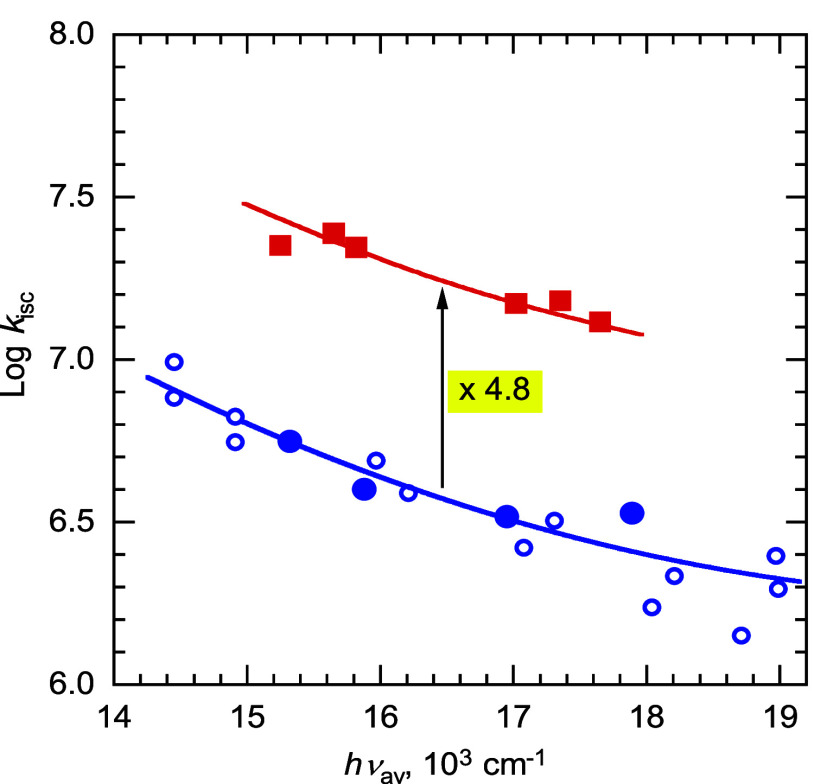
Plot of the logarithm of intersystem crossing rate constants, *k*_isc_, for cyanoanthracene exciplexes vs average emission energy, *h*ν_av_. Donors: alkylbenzenes (filled blue circles in benzene, Table 1; unfilled blue circles in other solvents, ref (65)) and alkoxybenzenes in benzene (red squares, Table 1).

**Figure 3:**
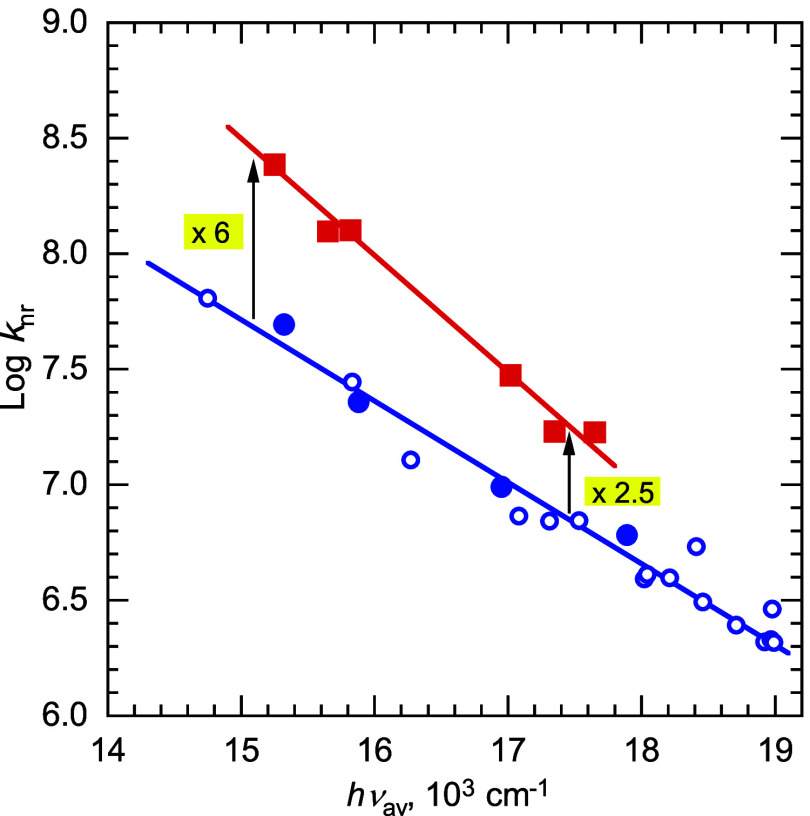
Plot of the logarithm of nonradiative decay rate constants, *k*_nr_, for cyanoanthracene exciplexes vs average emission energy, *h*ν_av_. Data point designations as in Figure 2.

The aim of the present work was to explore the effect, if any, of the type of substituents on electron donor moieties in closely related exciplex systems on the three rate constants in Scheme 1 and thus their impact on the fluorescence quantum yields. The acceptors used in the present study were 9,10-dicyanoanthracene (DCA), 2,9,10-tricyanoanthracene (TriCA), and 2,6,9,10-tetracyanoanthracene (TCA). Two classes of donors were investigated: alkylbenzenes (from *p*-xylene to hexamethylbenzene) and alkoxybenzenes (anisole, 4-methylanisole, and 3,4-dimethylanisole). Experiments were principally conducted in benzene as a solvent. The fluorescence quantum yields, Φ_f_, were measured by standard methods (see Methods). The intersystem crossing quantum yields, Φ_isc_, were measured as previously described.^63^ The nonradiative decay quantum yields, Φ_nr_, are given by (1 – Φ_f_ – Φ_isc_). The rate constants *k*_f_, *k*_isc_, and *k*_nr_ were obtained by dividing the corresponding quantum yields by the exciplex lifetimes, τ_Ex_, which were determined by time-correlated single photon counting (TCSPC). Average exciplex emission energies, *h*ν_av_, were calculated by fitting the spectra as previously described.^64^ The results are summarized in Table 1. Plots of the rate constants vs *h*ν_av_ are shown in Figures 1–3. Data in solvents other than benzene, mostly from previous work,^65^ are also included in Figures 2 and 3.

**Table 1:** Exciplexes of Cyanoanthracene Acceptors (A) with Alkyl- and Alkoxybenzene Donors (D) in Benzene: Average Emission Energies of Reduced Spectra (*h*ν_av_), Lifetimes (τ_Ex_), Fluorescence Quantum Yields (Φ_f_), Intersystem Crossing Quantum Yields (Φ_isc_), Nonradiative Quantum Yields (Φ_nr_ = 1 – Φ_f_ – Φ_isc_), and Rate Constants (*k*_f_, *k*_isc_, and *k*_nr_)

A^a^	D^b^	*h*ν_av_ (10^3^ cm^–1^)	τ_Ex_ (ns)	Φ_f_	Φ_isc_	Φ_nr_	*k*_f_ (10^6^ s^–1^)	*k*_isc_ (10^6^ s^–1^)	*k*_nr_ (10^6^ s^–1^)
DCA	HMB	18.01	80.0	0.371			4.64		
TCA	pXy	17.89	69.8	0.342	0.235	0.423	4.90	3.37	6.06
TCA	TMB	16.95	62.8	0.180	0.206	0.614	2.87	3.28	9.78
TCA	Dur	15.88	34.9	0.066	0.139	0.795	1.89	3.98	22.78
TCA	PMB	15.32	17.7	0.028	0.099	0.873	1.58	5.59	49.3
DCA	AN	19.88		0.439					
DCA	4MEAN	17.65	30.0	0.103	0.391	0.506	3.43	13.03	16.87
TriCA	AN	17.35	28.4	0.0874	0.43	0.483	3.08	15.14	16.99
DCA	34DMAN	17.02	21.2	0.0548	0.315	0.630	2.59	14.86	29.73
TriCA	4MEAN	15.82	6.65	0.0114	0.147	0.842	1.71	22.11	126.6
TCA	AN	15.65	6.63	0.0091	0.162	0.829	1.37	24.43	125
TriCA	34DMAN	15.25	3.75	0.0050	0.084	0.911	1.33	22.40	243

a Acceptors: 9,10-dicyanoanthracene (DCA), 2,9,10-tricyanoanthracene (TriCA), and 2,6,9,10-tetracyanoanthracene (TCA).

b Donors: alkyl-substituted benzenes: pXy (*p*-xylene), TMB (1,2,4-trimethylbenzene), Dur (durene), PMB (pentamethylbenzene), and HMB (hexamethylbenzene), and alkoxy-substituted benzenes: AN (anisole), 4MEAN (4-methylanisole), and 34DMAN (3,4-dimethylanisole).

**Figure 4:**
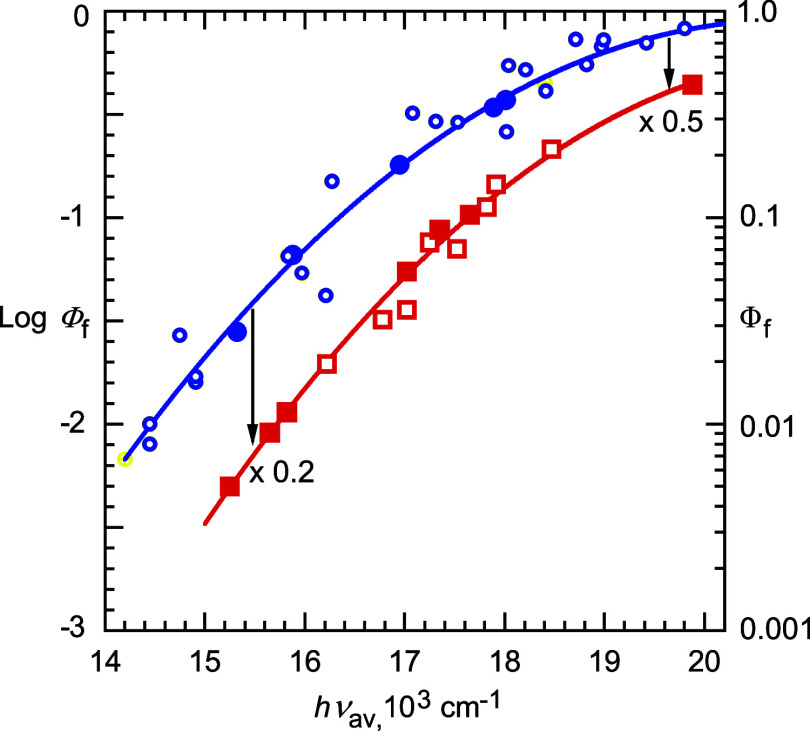
Exciplex fluorescence quantum yields, Φ_f_, vs average emission energies, *h*ν_av_. Filled data points as in Figures 1–3. Blue unfilled circles: alkylbenzenes in CHX, FB, dioxane, and TCE (from ref (65)). Red unfilled squares: alkoxybenzenes in CHX, FB, and TCE (Table 2).

**Figure 5:**
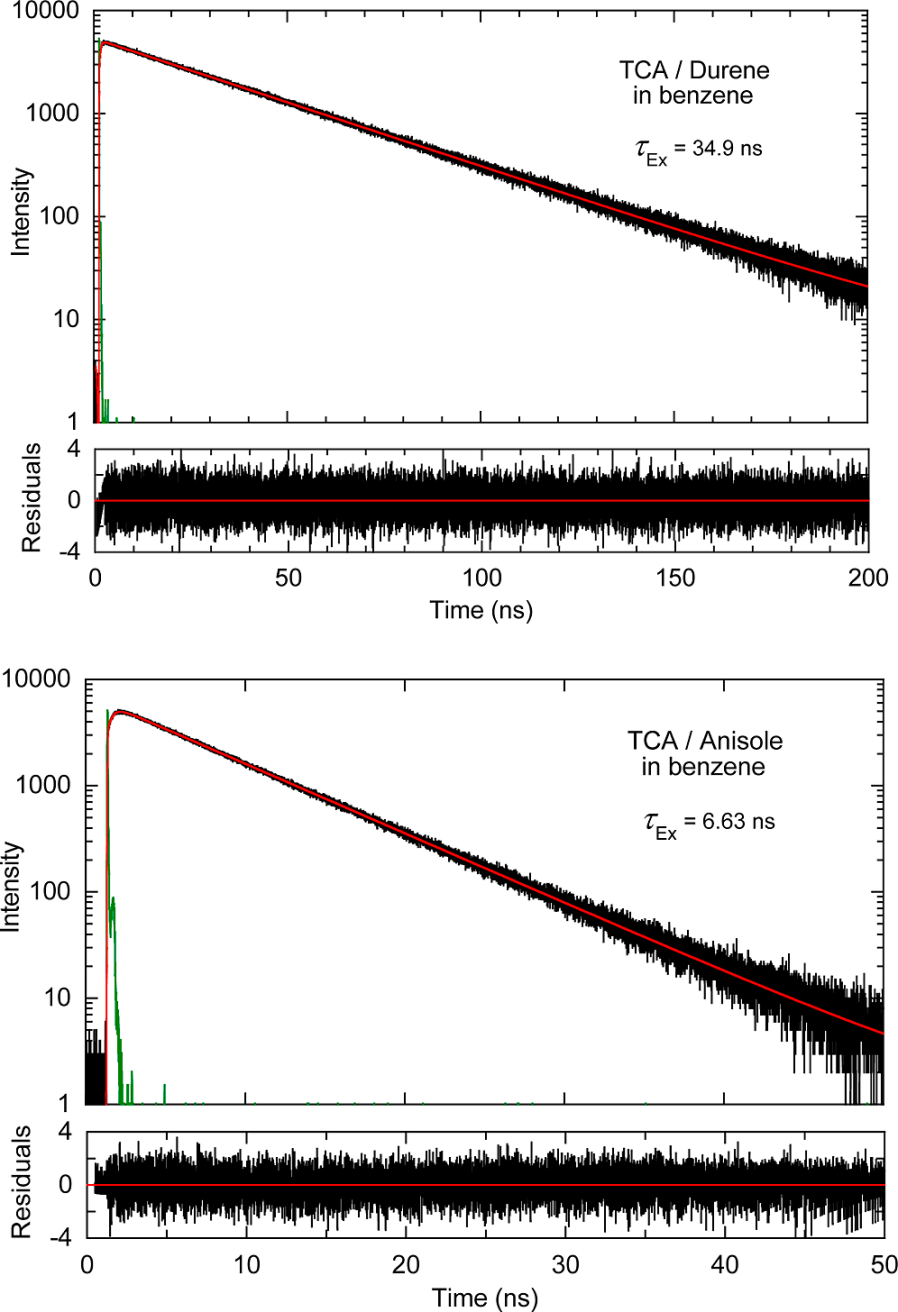
TCSPC measurements of TCA exciplexes in argon-purged benzene at room temperature monitored at 600 nm. Experimental data in black, best fit in red, and instrument response function (IRF) in green.

Whereas, at the same *h*ν_av_, *k*_f_ values of alkoxy-substituted donors are slightly (0.85 times) smaller than those of alkyl donors, *k*_isc_ values are on average 4.8 times larger and *k*_nr_ values are ∼2.5 times larger at the high *h*ν_av_ range and ∼6 times larger at the lower *h*ν_av_ range. The combined effect of these differences in rate constants between the two classes of donors is revealed by both a significant drop in fluorescence quantum yields of the alkoxy-substituted exciplexes, being 0.5 to 0.2 of those of alkyl donors at the same emission *h*ν_av_ (Figure 4), and shorter exciplex lifetimes for the alkoxy-substituted exciplexes (see, e.g., Figure 5).

We also measured the fluorescence quantum yields of exciplexes of alkoxy donors in several other low polarity solvents (Table 2). These and previously measured fluorescence quantum yields of several exciplexes of alkyl-substituted donors in these other solvents are shown in Figure 4, which demonstrates that the effect of alkyl vs alkoxy substituents on fluorescence quantum yields is not solvent-dependent.

**Table 2:** Fluorescence Quantum Yields (Φ_f_) for Exciplexes of Cyanoanthracene Acceptors (A) with Alkoxybenzene Donors (D) in Different Solvents and Average Emission Energies of Reduced Spectra (*h*ν_av_)^a^

A	D	solvent	*h*ν_av_ (10^3^ cm^–1^)	Φ_f_
DCA	34DMAN	CHX	18.47	0.214
TriCA	4MEAN	CHX	17.52	0.070_7_
TriCA	34DMAN	CHX	16.78	0.032
DCA	4MEAN	TCE	17.92	0.145
DCA	34DMAN	TCE	17.25	0.075_8_
TriCA	AN	TCE	17.82	0.112_4_
TriCA	4MEAN	TCE	16.22	0.019_5_
DCA	4MEAN	FB	17.02	0.035_7_

a Acceptors and donors as defined in Table 1. Solvents: cyclohexane (CHX), trichloroethylene (TCE), and fluorobenzene (FB).

**Figure 6:**
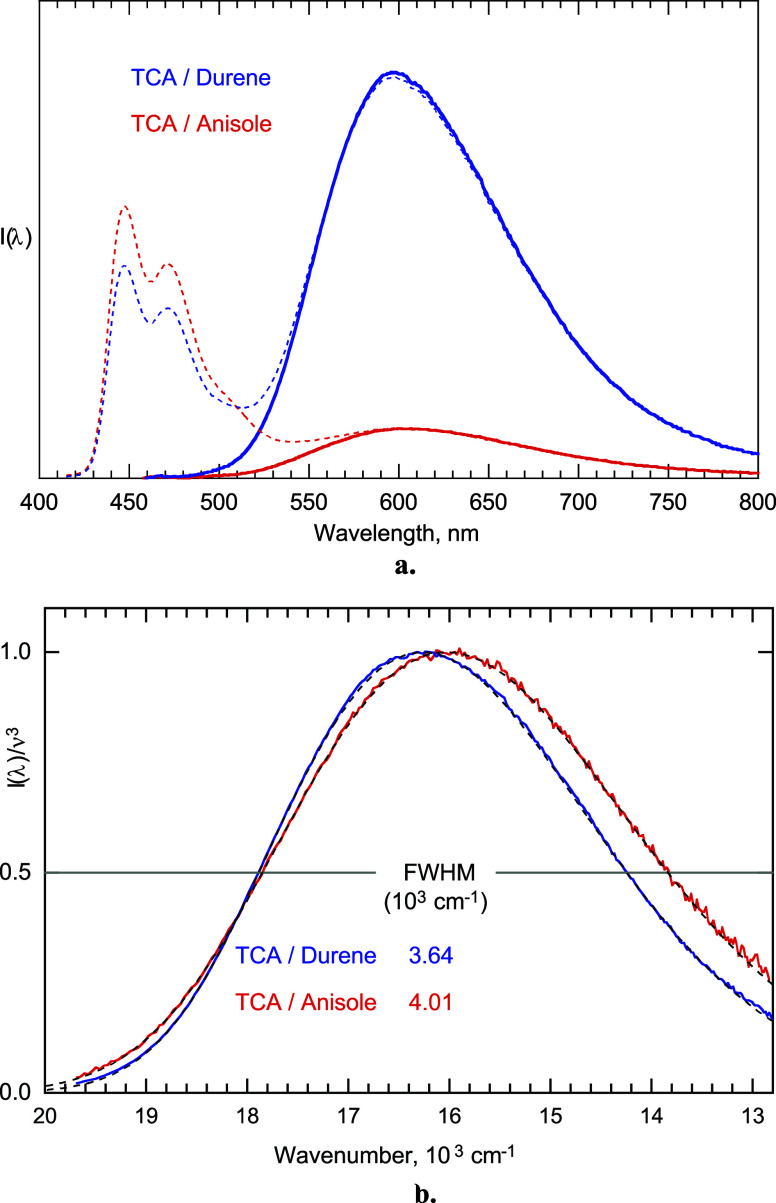
(a) Fluorescence spectra of TCA exciplexes with durene and anisole in benzene. Dashed curves: as measured with minor residual TCA fluorescence. Solid curves: after subtraction of the TCA fluorescence and correcting for incomplete interception of TCA*. (b) Reduced spectra of TCA exciplexes with durene and anisole in benzene.

Spectral distributions of exciplex fluorescence yield information about the reorganization energies associated with their decay. The full width at half-maximum (fwhm) of an exciplex spectrum increases with increasing reorganization energy, especially that associated with high vibrational modes. Shown in Figure 6 are reduced spectra^66,67^ of TCA exciplexes with durene and anisole. This and two other examples in the Supporting Information show that the fwhm of the alkoxy donor exciplexes is ca. 4000 cm^–1^ compared to ca. 3700 cm^–1^ for the alkyl donors. The larger reorganization energy of exciplexes of alkoxy donors compared to those of alkyl donors determined from emission spectra is in agreement with those estimated from MO calculations (see Supporting Information). The larger reorganization energies for the alkoxybenzene exciplexes are expected to lead to an increase in the nonradiative rate constants (*k*_nr_) for return electron transfer due to larger Franck–Condon overlap factors,^68^ consistent with the experimental results. Finally, we note that the plot of *k*_nr_ vs *h*ν_av_ for the alkylbenzene exciplexes has a somewhat smaller slope than for the alkoxybenzenes (Figure 3). This can be attributed to slightly larger relative increases in reorganization energies of the alkylbenzene donors with decreasing alkyl substituents, as previously described.^68^

The relative rate constants for fluorescence (*k*_f_) and intersystem crossing (*k*_isc_) for the alkoxybenzene vs alkylbenzene exciplexes can be understood in terms of differences in the electronic character of the donor radical cation moieties. Although the singly occupied molecular orbitals of both donors are expected to have largely π-character, the alkoxybenzenes have relatively low-lying nonbonding orbitals that can mix with the lower energy π states. This mixing will lead to an increase in *k*_isc_ due to greater spin–orbit coupling.^69^ Conversely, the mixing is expected to decrease *k*_f_.^69^

## 4 Conclusions

The results described herein show that the lower fluorescence quantum yields (Φ_f_) for exciplexes containing alkoxy- vs alkyl-substituted arene electron donors are due to the combined effect of smaller radiative rate constants and larger rate constants for nonradiative decay (*k*_nr_) and intersystem crossing (*k*_isc_). These results have obvious implications for the rational choice of electron donor moieties to increase quantum yields for exciplex emission.^70,71^ Doubtless, similar studies for exciplexes using other heteroatom-substituted electrons could be of utility when designing more efficient light-emitting exciplex systems.

## Supporting Information Available

Spectrum of DCA/anisole in benzene, fwhm of the exciplex spectra of alkyl- vs alkoxybenzene donors, calculated electron transfer reorganization energies, calculated geometries and energies, and references (PDF)
