# Supplementary material for: Effect of Alkyl- vs Alkoxy-Arene Substituents on the Deactivation Processes and Fluorescence Quantum Yields of Exciplexes
Source: J Phys Chem A. 2024 Dec 13;128(51):10986–92. doi: 10.1021/acs.jpca.4c06279 (PMC11684015; doi:10.1021/acs.jpca.4c06279)
Supplement: Supplementary file 1 — jp4c06279_si_001.pdf [file jp4c06279_si_001.pdf]

Supporting Information

Effect of Alkyl- vs Alkoxy-Arene Substituents on the Deactivation Processes and Fluorescence Quantum Yields of Exciplexes

Joseph P. Dinnocenzo,* Olesya Haze, Cavan Fleming, and Samir Farid*

Department of Chemistry, University of Rochester, Rochester, New York 14627, USA

Table of Contents	Page
1. Spectrum of DCA/Anisole in Benzene	S2
2. FWHM of Exciplex Spectra of Alkyl vs Alkoxy Benzene Donors	S3
3. Calculated Electron Transfer Reorganization Energies	S4
4. Calculated Geometries and Energies	S5
5. References	S13

1. Spectrum of DCA/Anisole in Benzene

The exciplex of DCA/anisole in benzene is formed reversibly. As a result, even at anisole concentration of 0.5 M, only ~40% of excited DCA is quenched, Figure S1.

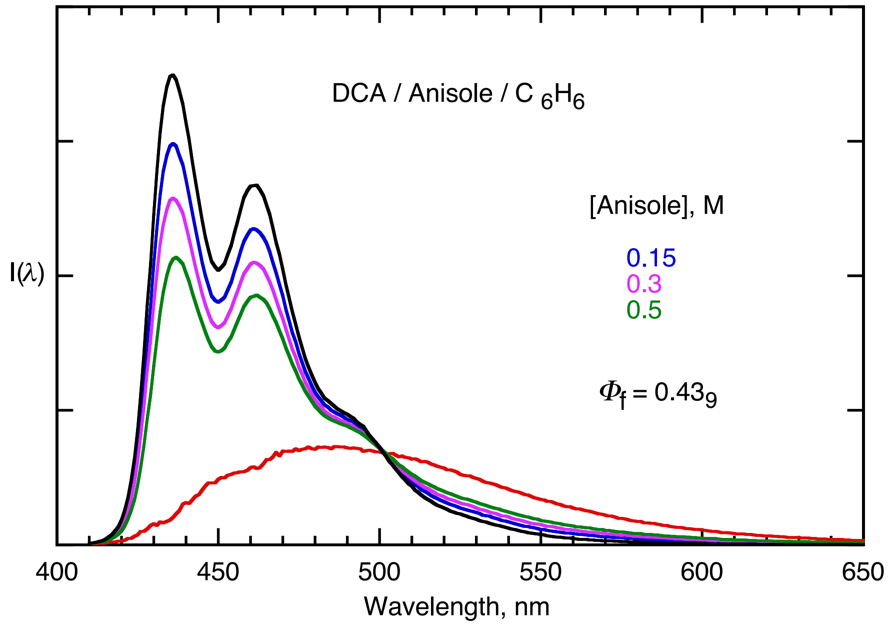

Figure S1. Fluorescence spectra of DCA/anisole in benzene. The black curve: DCA in absence of anisole. Red curve: Averaged exciplex spectrum obtained by subtracting residual DCA fluorescence and correcting for incomplete interception.

2. FWHM of Exciplex Spectra of Alkyl- vs Alkoxy-Benzene Donors

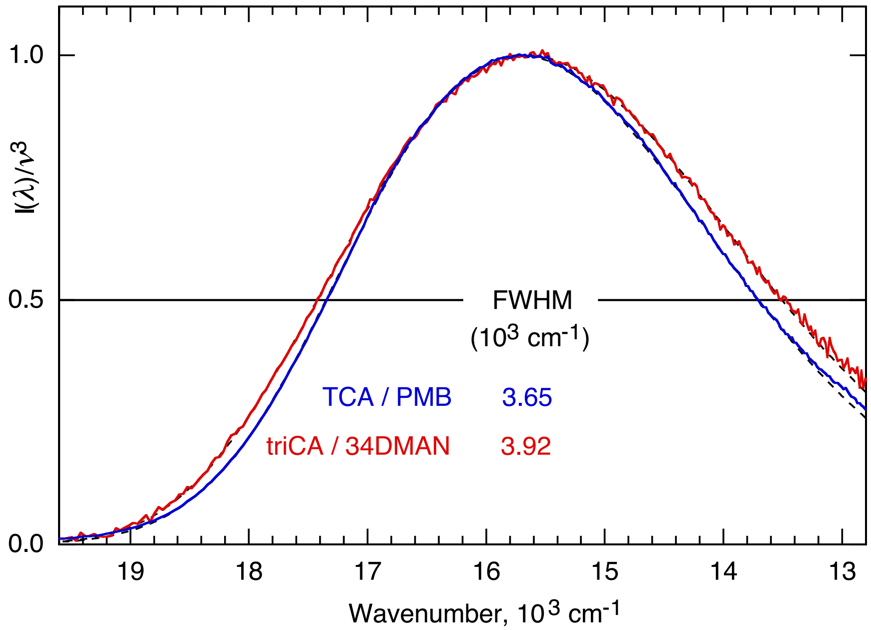

Figure S2. Reduced exciplex spectra of TCA/pentamethylbenzene and of TriCA/3,4-dimethylanisole in benzene.

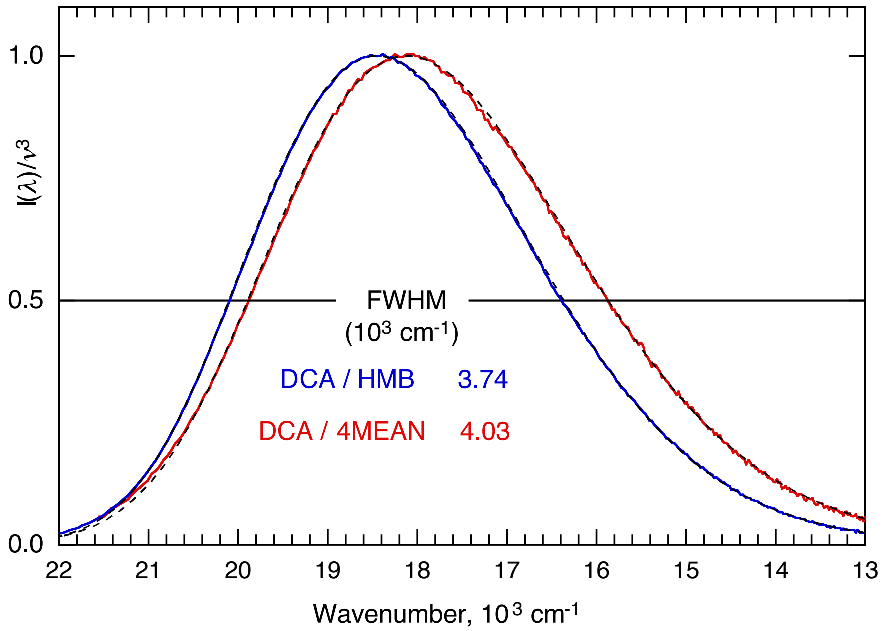

Figure S3. Reduced exciplex spectra of DCA/hexamethylbenzene and of DCA/4-methylanisole in benzene.

3. Calculated Electron Transfer Reorganization Energies

Due to the similarity in their oxidation potentials, the electron transfer reorganization energies (λ_v) for anisole (1.773 V vs. SCE in CH_3CN)[S1] and durene (1.753 V vs. SCE in CH_3CN)[S2] were calculated by the method of Nelsen et al.[S3] At the M06/6-311+G(2df,2p) level of theory the resulting reorganization energies were 0.43 and 0.31 eV, respectively. The results from the underlying calculations are summarized below.

Molecule	Energy (au)	λ_v
Anisole	–346.628134	
Ansole$^{+\bullet}$	–346.330884	
Anisole in cation geometry	–346.620111	
Anisole$^{+\bullet}$ in neutral geometry	–346.323084	
		0.43 eV
Durene	–389.315388	
Durene$^{+\bullet}$	–389.029461	
Durene in cation geometry	–389.309647	
Durene$^{+\bullet}$ in neutral geometry	–389.023816	
		0.31 eV

4. Calculated Geometries and Energies

Anisole

E[M06/6-311+G(2df,2p)] = –346.628134 au
zero imaginary frequencies

ATOM		Coordinates (Angstroms) X	Y	Z
1	C	-0.4163443749	0.0000294552	-0.369610262
2	C	0.2325424805	-0.0000146832	2.3370244453
3	C	0.9155284221	0.0000325788	0.0244221168
4	C	-1.4223201683	0.0000139177	0.5934420537
5	C	-1.0963355635	-0.0000067289	1.9328933073
6	C	1.2268819543	0.0000036373	1.3777737329
7	H	1.7131440793	0.0000487039	-0.7064061169
8	H	-2.4536454105	0.0000130256	0.2612299097
9	H	-1.8880885626	-0.0000207355	2.6725492045
10	H	2.2685938975	-0.0000061751	1.6768292715
11	H	0.4859497538	-0.0000362719	3.3895498050
12	O	-0.8323841157	0.0000300403	-1.6554770755
13	C	0.1343382847	-0.0000159701	-2.6700888617
14	H	0.7694629581	0.8907980323	-2.6256578000
15	H	-0.4067566454	-0.0000360614	-3.6128719321
16	H	0.7694330104	-0.8908327649	-2.6256017977

Anisole$^{+\bullet}$

E[M06/6-311+G(2df,2p)] = –346.330884 au
zero imaginary frequencies

ATOM		X	Y	Z
		Coordinates (Angstroms)		
1	C	-0.3871221757	0.0008749800	-0.3630608721
2	C	0.2206376414	-0.0014323169	2.3289994950
3	C	0.9762198894	0.0037460021	0.0397664005
4	C	-1.4371496664	0.0000223572	0.6088639651
5	C	-1.1264506824	-0.0015900163	1.9338749345
6	C	1.2601296822	0.0011767506	1.3709363410
7	H	1.7683368355	0.0077380507	-0.6957894581
8	H	-2.4572036337	0.0015133889	0.2454518927
9	H	-1.9083552027	-0.0010447786	2.6810871742
10	H	2.2893811540	0.0023632388	1.7051250992
11	H	0.4723097780	-0.0026630819	3.3834288932
12	O	-0.7859586893	-0.0033538105	-1.5924291666
13	C	0.1260929145	-0.0023483415	-2.7031460696
14	H	0.7422091865	0.8962979797	-2.6754977267
15	H	-0.4966884969	-0.0012184855	-3.5910306577
16	H	0.7436114659	-0.9000819169	-2.6765802445

Anisole in cation geometry
E[M06/6-311+G(2df,2p)] = –346.620111 au
single point calculation

ATOM		X	Y	Z
		Coordinates (Angstroms)		
1	C	-0.3871221757	0.0008749800	-0.3630608721
2	C	0.2206376414	-0.0014323169	2.3289994950
3	C	0.9762198894	0.0037460021	0.0397664005
4	C	-1.4371496664	0.0000223572	0.6088639651
5	C	-1.1264506824	-0.0015900163	1.9338749345
6	C	1.2601296822	0.0011767506	1.3709363410
7	H	1.7683368355	0.0077380507	-0.6957894581
8	H	-2.4572036337	0.0015133889	0.2454518927
9	H	-1.9083552027	-0.0010447786	2.6810871742
10	H	2.2893811540	0.0023632388	1.7051250992
11	H	0.4723097780	-0.0026630819	3.3834288932
12	O	-0.7859586893	-0.0033538105	-1.5924291666
13	C	0.1260929145	-0.0023483415	-2.7031460696
14	H	0.7422091865	0.8962979797	-2.6754977267
15	H	-0.4966884969	-0.0012184855	-3.5910306577
16	H	0.7436114659	-0.9000819169	-2.6765802445

Anisole$^{+•}$ in neutral geometry

E[M06/6-311+G(2df,2p)] = –346.323084 au

single point calculation

ATOM		Coordinates (Angstroms) X	Y	Z
1	C	-0.4163443749	0.0000294552	-0.369610262
2	C	0.2325424805	-0.0000146832	2.3370244453
3	C	0.9155284221	0.0000325788	0.0244221168
4	C	-1.4223201683	0.0000139177	0.5934420537
5	C	-1.0963355635	-0.0000067289	1.9328933073
6	C	1.2268819543	0.0000036373	1.3777737329
7	H	1.7131440793	0.0000487039	-0.7064061169
8	H	-2.4536454105	0.0000130256	0.2612299097
9	H	-1.8880885626	-0.0000207355	2.6725492045
10	H	2.2685938975	-0.0000061751	1.6768292715
11	H	0.4859497538	-0.0000362719	3.3895498050
12	O	-0.8323841157	0.0000300403	-1.6554770755
13	C	0.1343382847	-0.0000159701	-2.6700888617
14	H	0.7694629581	0.8907980323	-2.6256578000
15	H	-0.4067566454	-0.0000360614	-3.6128719321
16	H	0.7694330104	-0.8908327649	-2.6256017977

Durene

E[M06/6-311+G(2df,2p)] = –389.315388 au
zero imaginary frequencies

ATOM		Coordinates (Angstroms) X	Y	Z
1	H	-0.0001905068	-0.0000552815	-2.4501294296
2	C	0.0003488314	-0.0000370481	-1.3629070682
3	C	0.0015768427	-0.0000224728	1.3629830127
4	C	0.0003857330	-1.2186684948	-0.6982371197
5	C	0.0005670044	1.2185940610	-0.6982827299
6	C	0.0012345952	1.2185993917	0.6983075136
7	C	0.0010587444	-1.2186641833	0.6983560216
8	H	0.0020324316	-0.0000051389	2.4502053206
9	C	-0.0011150645	2.5017073237	-1.4652004658
10	H	0.8714010698	3.1170558437	-1.2274974851
11	H	-0.8805076117	3.1096494329	-1.2345041535
12	H	0.0035854586	2.3200093782	-2.5400876552
13	C	0.0010391114	2.5017749731	1.4651221529
14	H	0.8773882561	3.1133112098	1.2315143129
15	H	-0.0000409382	2.3201175068	2.5401700321
16	H	-0.8746321188	3.1134885177	1.2300890078
17	C	0.0010786421	-2.5017461623	1.4653219247
18	H	0.8792771073	-3.1117844040	1.2341109019
19	H	-0.8729361751	-3.1150750627	1.2281516289
20	H	-0.0031102877	-2.3199013325	2.5403224392
21	C	-0.0014851842	-2.5017610660	-1.4652183653
22	H	0.8705086231	-3.1174185779	-1.2272346270
23	H	0.0039704069	-2.3198617831	-2.5401140888
24	H	-0.8814349709	-3.1093066307	-1.2352411867

Durene$^{+\bullet}$

E[M06/6-311+G(2df,2p)] = –389.029461 au
zero imaginary frequencies

ATOM		Coordinates (Angstroms) X	Y	Z
1	H	-0.0026120100	-0.0002473042	-2.4799866201
2	C	-0.0018537996	-0.0002430565	-1.3958444788
3	C	0.0028782688	-0.0003532632	1.3958004649
4	C	-0.0007137454	-1.2107319893	-0.7260348328
5	C	-0.0005929685	1.2100487806	-0.7258815333
6	C	0.0013337436	1.2099013758	0.7258743482
7	C	0.0007192699	-1.2106511037	0.7259751871
8	H	0.0043532924	-0.0003705027	2.4799424369
9	C	-0.0004912379	2.4867043149	-1.4659359986
10	H	0.8718632710	3.0933708269	-1.2015336790
11	H	-0.8722555091	3.0928613152	-1.2005958578
12	H	-0.0007611844	2.3322548041	-2.5420508155
13	C	0.0001372487	2.4865450914	1.4659156610
14	H	0.8720430867	3.0928502038	1.2022901696
15	H	-0.0012008514	2.3327441703	2.5425597866
16	H	-0.8723045531	3.0921912346	1.1999730905
17	C	-0.0000810730	-2.4867421217	1.4674005807
18	H	0.8733939195	-3.0922348142	1.2066194557
19	H	-0.8714471581	-3.0942709299	1.2026246783
20	H	-0.0028431770	-2.3300212634	2.5440388162
21	C	0.0002026428	-2.4864436799	-1.4679353672
22	H	0.8689488858	-3.0965978955	-1.1995385347
23	H	0.0064754000	-2.3301589021	-2.5435688963
24	H	-0.8751917617	-3.0904052912	-1.2101081672

Durene in cation geometry
E[M06/6-311+G(2df,2p)] = –389.309647 au
single point calculation

ATOM		Coordinates (Angstroms) X	Y	Z
1	H	-0.0026120100	-0.0002473042	-2.4799866201
2	C	-0.0018537996	-0.0002430565	-1.3958444788
3	C	0.0028782688	-0.0003532632	1.3958004649
4	C	-0.0007137454	-1.2107319893	-0.7260348328
5	C	-0.0005929685	1.2100487806	-0.7258815333
6	C	0.0013337436	1.2099013758	0.7258743482
7	C	0.0007192699	-1.2106511037	0.7259751871
8	H	0.0043532924	-0.0003705027	2.4799424369
9	C	-0.0004912379	2.4867043149	-1.4659359986
10	H	0.8718632710	3.0933708269	-1.2015336790
11	H	-0.8722555091	3.0928613152	-1.2005958578
12	H	-0.0007611844	2.3322548041	-2.5420508155
13	C	0.0001372487	2.4865450914	1.4659156610
14	H	0.8720430867	3.0928502038	1.2022901696
15	H	-0.0012008514	2.3327441703	2.5425597866
16	H	-0.8723045531	3.0921912346	1.1999730905
17	C	-0.0000810730	-2.4867421217	1.4674005807
18	H	0.8733939195	-3.0922348142	1.2066194557
19	H	-0.8714471581	-3.0942709299	1.2026246783
20	H	-0.0028431770	-2.3300212634	2.5440388162
21	C	0.0002026428	-2.4864436799	-1.4679353672
22	H	0.8689488858	-3.0965978955	-1.1995385347
23	H	0.0064754000	-2.3301589021	-2.5435688963
24	H	-0.8751917617	-3.0904052912	-1.2101081672

Durene$^{+\bullet}$ in neutral geometry
E[M06/6-311+G(2df,2p)] = –389.023816 au
single point calculation

ATOM		Coordinates (Angstroms) X	Y	Z
1	H	-0.0001905068	-0.0000552815	-2.4501294296
2	C	0.0003488314	-0.0000370481	-1.3629070682
3	C	0.0015768427	-0.0000224728	1.3629830127
4	C	0.0003857330	-1.2186684948	-0.6982371197
5	C	0.0005670044	1.2185940610	-0.6982827299
6	C	0.0012345952	1.2185993917	0.6983075136
7	C	0.0010587444	-1.2186641833	0.6983560216
8	H	0.0020324316	-0.0000051389	2.4502053206
9	C	-0.0011150645	2.5017073237	-1.4652004658
10	H	0.8714010698	3.1170558437	-1.2274974851
11	H	-0.8805076117	3.1096494329	-1.2345041535
12	H	0.0035854586	2.3200093782	-2.5400876552
13	C	0.0010391114	2.5017749731	1.4651221529
14	H	0.8773882561	3.1133112098	1.2315143129
15	H	-0.0000409382	2.3201175068	2.5401700321
16	H	-0.8746321188	3.1134885177	1.2300890078
17	C	0.0010786421	-2.5017461623	1.4653219247
18	H	0.8792771073	-3.1117844040	1.2341109019
19	H	-0.8729361751	-3.1150750627	1.2281516289
20	H	-0.0031102877	-2.3199013325	2.5403224392
21	C	-0.0014851842	-2.5017610660	-1.4652183653
22	H	0.8705086231	-3.1174185779	-1.2272346270
23	H	0.0039704069	-2.3198617831	-2.5401140888
24	H	-0.8814349709	-3.1093066307	-1.2352411867

REFERENCES

S1. Luo, P.; Feinberg, E. C.; Guirado, G.; Farid, S.; Dinnocenzo, J. P. Accurate Oxidation Potentials of Forty Benzene and Biphenyl Derivatives with Heteroatom Substituents. *J. Org. Chem.* **2014**, *79*, 9297–9304.

S2. Nanosecond Redox Equilibrium Method for Determining Oxidation Potentials in Organic Media". Guirado, G.; Fleming, C. N.; Lingenfelter, T. G.; Williams, M. L.; Zuilhof, H.; Dinnocenzo, J. P. *J. Am. Chem. Soc.* **2004**, *126*, 14086–14094.

S3. Nelsen, S. F.; Blackstock, S. C.; Kim, Y. Estimation of Inner Shell Marcus Terms for Amino Nitrogen Compounds by Molecular Orbital Calculations. *J. Am. Chem. Soc.* **1987**, *109*, 677−682.
